# Spermidine supplementation influence on protective enzymes of *Apis mellifera* (Hymenoptera: Apidae)

**DOI:** 10.1093/jisesa/ieae098

**Published:** 2024-10-09

**Authors:** Tatjana V Čelić, Srđana Đorđievski, Elvira L Vukašinović, Ivan Pihler, Danijela Kojić, Jelena Purać

**Affiliations:** Department of Biology and Ecology, Faculty of Sciences, University of Novi Sad, Novi Sad, Serbia; Department of Biology and Ecology, Faculty of Sciences, University of Novi Sad, Novi Sad, Serbia; Department of Biology and Ecology, Faculty of Sciences, University of Novi Sad, Novi Sad, Serbia; Department of Animal Science, Faculty of Agriculture, University of Novi Sad, Novi Sad, Serbia; Department of Biology and Ecology, Faculty of Sciences, University of Novi Sad, Novi Sad, Serbia; Department of Biology and Ecology, Faculty of Sciences, University of Novi Sad, Novi Sad, Serbia

**Keywords:** polyamine, nutrition, antioxidative protection, immunity

## Abstract

Dietary supplementation has been proposed as a sustainable way to improve the health and resilience of honey bees (*Apis mellifera*, L.), as the decline in their numbers in recent decades has raised scientific, environmental, and economic concerns. Spermidine, a natural polyamine, has been shown to be a promising substance for honey bee supplementation, as its health-promoting effects have been demonstrated in numerous studies and in different organisms. As already shown, supplementation with spermidine at a certain concentration prolonged lifespan, reduced oxidative stress, and increased antioxidative capacity in honey bees. The aim of the present study was to investigate whether spermidine supplementation affects gene expression and/or enzyme activity of antioxidative and detoxification enzymes and immune response markers in honey bee workers. The different gene expression and enzyme activity patterns observed in abdominal and head tissues in response to spermidine supplementation suggest tissue-specific and concentration-dependent effects. In addition, the immune response markers suggest that spermidine has the ability to boost honey bee immunity. The observed changes make a valuable contribution to understanding the molecular mechanisms by which spermidine may exert its beneficial effects on the bee’s health and lifespan. These results support the idea of the use of spermidine supplementation to promote bee health and resilience to environmental stressors, emphasizing that the dose must be carefully chosen to achieve a balance between the pro- and antioxidant effects of spermidine.

## Introduction

Honey bee (*Apis mellifera* L.) is one of the world’s most important pollinators, so the decline in its number in recent decades has raised scientific, environmental, and economic concerns (Potts et al. 2010). Several factors, acting individually and synergistically, are responsible for this decline, including extensive use of agrochemicals, habitat destruction, climate change, but also the improper management of apiaries (Cox-Foster et al. 2007, Potts et al. 2010). At the molecular level, these factors activate numerous signaling pathways, and some of the most important mechanisms that negatively impact bee health and survival are attributed to the promotion of oxidative stress (Morimoto et al. 2011, Nikolić et al. 2015, 2016, Simone-Finstrom et al. 2016, Orčić et al. 2017). The generation of oxidative stress can lead to macromolecular damage and impaired immunocompetence (Brandt et al. 2016, Polykretis et al. 2016, Orčić et al. 2022), making honey bees more susceptible to pathogens.

Honey bees, like many other insect species, are particularly susceptible to oxidative stress due to their very active lifestyle (Korayem and Khodairy 2012), as oxygen demand is extremely high during insect flight (Krogh and Weis-fogh 1951). In addition, nectar and pollen consumed by honey bees contain various allelochemicals, including phenols. These compounds, while beneficial in some contexts, can undergo metabolic oxidation and generate reactive oxygen species (ROS) (Ahmad 1992, Mittapalli et al. 2007). When present in high concentrations, ROS interact with biomolecules such as lipids, proteins, and DNA, oxidizing them and thereby irreversibly altering normal cellular function (Sharma et al. 2012). Furthermore, after the honey bee genome sequence was published in 2006, significant differences were observed compared to the previously sequenced genomes of the fruit fly, *Drosophila melanogaster*, and the malaria mosquito, *Anopheles gambiae* (The Honeybee Genome Sequencing Consortium 2006). One of the observed differences is a smaller number of genes for immunity and enzymes involved in detoxification, which may result in the greater sensitivity of bees to diseases and xenobiotics (Zhao and Liu 2022). This fact becomes more important since bees are highly exposed to pollutants in the environment due to their direct contact with plants when collecting pollen and nectar, especially pesticides, which are increasingly used in agriculture (Krupke et al. 2012). Moreover, due to the lack of genes for enzymes related to the metabolism of xenobiotics, honey bees are particularly susceptible to oxidative stress caused by the incomplete microsomal oxidation of xenobiotics such as insecticides (Badiou-Bénéteau et al. 2012), so the antioxidant protection system may play an important defensive role in the presence of environmental stressors. Compared to the known genomes of other insects, the genome of *A. mellifera* encodes fewer immune proteins involved in the process of immune response, from pathogen recognition to immune effectors, implying less flexibility in the abilities of honeybees to detect and resist pathogens (Zhao and Liu 2022). Honey bees rely on social behaviors such as grooming to complement their innate immunity. However, these behaviors cannot fully compensate for the reduced genetic flexibility in immune response, especially when faced with novel or rapidly evolving pathogens (Richard et al. 2008). Therefore, strengthening the existing components of the immune response and antioxidative defense system should be a priority in improving honey bee health.

With this in mind, nutritional supplementation has been proposed as a sustainable way to improve the health and resilience of honey bees (Stevanovic et al. 2018, Hendriksma et al. 2019, Geldert et al. 2021). Spermidine, a natural polyamine, is a promising substance for honey bee nutritional supplementation, as its health-promoting effects have been demonstrated in numerous studies and in various organisms (Madeo et al. 2018, Hirano et al. 2021). As a polycation, spermidine interacts with negatively charged molecules and regulates important cellular functions (Kaeberlein 2009, Minois 2014), however, its exact role is still unclear. Many studies in both animal model organisms and humans have reported that polyamines, especially spermidine, are closely associated with antiaging functions. Namely, endogenous spermidine levels decrease in an age-dependent manner (Eisenberg et al. 2009, Pucciarelli et al. 2012, Gupta et al. 2013), suggesting a link between endogenous spermidine concentration and age-related impairments (Madeo et al. 2019). The most important mechanism of spermidine action is autophagy (Morselli et al. 2009). However, there are other mechanisms for which it has not yet been formally established whether they are completely independent of autophagy, including the antioxidant effect (Rider et al. 2007, Minois et al. 2012, Madeo et al. 2018) and immunostimulating effect of this molecule (Madeo et al. 2018, Al-Habsi et al. 2022).

As previously shown, dietary supplementation with spermidine at a certain concentration prolonged lifespan, reduced oxidative stress, and increased antioxidative capacity in honey bees (Đorđievski et al. 2023). The aim of the present study was to investigate whether dietary supplementation with spermidine affects gene expression and/or enzyme activity of antioxidative enzymes superoxide dismutase (SOD) and catalase (CAT), transcription factor Cap’n’collar (*Cnc*), detoxification enzyme glutathione *S*-transferase (GST). Activities of SOD and CAT are functionally connected and represent the front-line defense against oxidative stress. SOD catalyzes the dismutation of superoxide anion free radical during which hydrogen peroxide is formed, which is a substrate for CAT. SOD exists in three isoforms with different localizations in the organism: the cytoplasmic Cu/ZnSOD (SOD1), the mitochondrial MnSOD (SOD2), and the extracellular Cu/ZnSOD (SOD3) (Fukai and Ushio-Fukai 2011). GST is an enzyme known for its crucial role in detoxification processes, facilitating the conjugation of glutathione with various electrophilic substances. Notably, there are different isoforms of GST, each with specific functions, contributing to the diverse roles of this enzyme in cellular defense mechanisms. The Cap’n’collar transcription factor family consists of a group of proteins involved in various biological processes, including cellular response to oxidative stress (Sykiotis and Bohmannn 2010, Pitoniak and Bohmann 2015).

Furthermore, to investigate whether spermidine supplementation affects the immunity of honey bees, main cellular immunity markers phenoloxidase (PO), prophenoloxidase (ProPO), and phenoloxidase-activating factor 2 (*Ppaf2*) were analyzed. The expression of the *Ppo* gene leads to the synthesis of prophenoloxidase, a zymogenic form of the enzyme that is activated by specific proteolytic cleavage by serine proteases that constitute the prophenoloxidase activating system, with *Ppaf2* being part of it (Mak and Saunders 2006). Higher PO activity is an indicator of better condition of the individual, as PO activity usually decreases after exposure to pesticides (Orčić et al. 2022) and pathogens (Eleftherianos et al. 2007, Ullah et al. 2014).

## Materials and Methods

### Experimental Setup

The honey bees used for the experiment were taken from experimental hives located at the Fruška Gora mountain (45°22’ N; 19°53’ E), near Novi Sad, Serbia. The hives were inspected to determine the health status of the worker bees, and it was concluded that the colonies were healthy and that there were no visible signs of infectious disease. The colonies had not been exposed to any chemicals to treat diseases for at least 10 months before the start of the experiment. Two frames with brood were transferred from the hive to a smaller glass observation hive, which was kept in the incubator under controlled conditions, in the dark at 34 °C, mimicking the conditions in the hive, to allow the worker bees to hatch. After 24 h, the temperature was lowered to 28 °C and the brood frames were replaced with frames with honey and bee bread to provide food for the young bees. Five days after emergence, the individual bees were randomly transferred to experimental 2 L plastic boxes and returned to the incubator. Each box contained about 35 bees. Small holes in the boxes ensured even ventilation and the bees were fed with a plastic syringe with a cut-off tip, which was pulled into the box and fixed with adhesive tape.

Bees were fed with 50% (w/v) sucrose as the basic feed solution (control group), with the addition of 0.1 and 1 mM spermidine, to form S_0.1_ and S_1_ experimental groups, respectively. The studied concentrations were determined after the experiment with a wide range of spermidine concentrations, and the concentrations that showed a positive effect on survival and lifespan were selected (Đorđievski et al. 2023). Each experimental group consisted of 3 biological replicates. Fresh feed solutions supplemented with spermidine were prepared each day from 1 M stock solution, made from synthetic spermidine (Sigma-Aldrich Chemie GmbH). Each day the amount of eaten feed solution was measured, the dead bees were removed, and their number was recorded. The experiment lasted 17 days. After that, the remaining live bees were immediately frozen and stored at −80 °C for further analysis.

### Enzymatic Assays

The activity of the antioxidant enzyme was determined in 10% (w/v) homogenates of honey bee head and abdomen in 50 mM phosphate buffer, pH 7.4. For each replicate, 5 heads/abdomens from worker bees were homogenized in the ice-cold buffer; the crude homogenates were centrifuged at 10,000 g (4 °C) for 10 min, the supernatant was transferred to clean tubes and stored at −20 °C until enzyme assays were performed. The activity of SOD was measured spectrophotometrically at 550 nm as the inhibition of reduction of cytochrome C by superoxide radicals generated in the xanthine-xanthine oxidase reaction (McCord and Fridovich 1968), while the activity of CAT was measured spectrophotometrically as the rate of hydrogen peroxide degradation at 240 nm based on the method by Aebi (1984). Glutathione *S*-transferase was determined spectrophotometrically by monitoring the formation of the product of the reaction between reduced glutathione and 1-chloro-2,4-dinitrobenzene at 340 nm (Habig et al. 1974). Phenoloxidase activity was determined in the abdomen of honey bees according to the protocol published by Laughton and Siva-Jothy (2011), as the main immune response takes place in this body region (Lin et al. 2022). All assays are described in more detail by Orčić et al. (2022). Protein concentration was determined using the Bradford method (1976), with bovine serum albumin (Bio-Rad) as protein standard. The specific activity of the enzymes was expressed as the number of units of enzyme activity per milligram of protein.

### Relative Gene Expression (qPCR)

Total RNA used to analyze relative gene expression was extracted from the head and abdomen of honey bees. Three heads/abdomens were pooled for each biological replicate. Total RNA was extracted with RNA Extracol (EurX) according to the manufacturer’s protocol and diluted in 30–50 μl RNase/DNase-free water (Sigma-Aldrich). The RNA concentration was measured at 260 nm using the BioSpec-nano spectrophotometer (Shimadzu), and the purity of total RNA was determined using the 260/280 absorbance ratio. After determination of purity and concentration, total RNA was diluted to 500 ng/μl and stored at − 80 °C. The cDNA was synthesized using the QuantiTect Reverse Transcription Kit (Qiagen) according to the manufacturer’s protocol, starting with 1 μg total RNA. The obtained samples were stored at − 20 °C.

Relative expression was measured for 7 antioxidant system genes, Cap’n’collar (*Cnc*), Cu, Zn-superoxide dismutase (*Sod1*), Mn-superoxide dismutase (*Sod2*), catalase (*Cat)*, glutathione *S*-transferase delta class (*GstD1*), glutathione *S*-transferase sigma class (*GstS1*), and glutathione *S*-transferase microsomal class (*Gstmic1*), as well as for genes for prophenoloxidase (*ProPo*) and phenoloxidase-activating factor 2 (*Ppaf2*). Genes for β-actin (*ActB*) and ribosomal protein 49 (*Rp49*) were used as an endogenous control. The suitability of these honey bee genes as endogenous controls in qPCR assays has already been confirmed (Lourenço et al. 2008). The sequences of the PCR primers are listed in Supplementary Table S1. The reaction in a total volume of 14 μl included 7 μl of 2 × SYBR Green PCR Master Mix (Applied Biosystems), 500 nM of each primer, and 40 ng of cDNA. Quantitative PCR of the cDNA products was performed using the MasterCycler RealPlex4 (Eppendorf). The amplification program consisted of an initial preincubation step at 95 °C (10 min) and 40 cycles of 95 °C (15 s) and 60 °C (1 min) with an additional step for melting curve analysis to confirm amplification of a single-gene product.

### Data Analysis

Enzyme activity data were analyzed using STATISTICA software (TIBCO Software Inc. 2020). Data Science Workbench, version 14, with one-way ANOVA followed by Dunnet post-hoc test and expressed as mean ± SEM. A significant difference between each experimental group and control was estimated with a confidence interval of *P* ≤ 0.05. The difference in gene expression was calculated using the software REST 2009 (Qiagen), with relative up- or downregulations calculated and tested for statistical significance between control and experimental groups with *P* ≤ 0.05 confidence interval using the integrated Bootstrap randomization test (2,000 iterations) (Pfaffl et al. 2002).

## Results

### Antioxidant Enzymes Activity

**Fig. 1.**
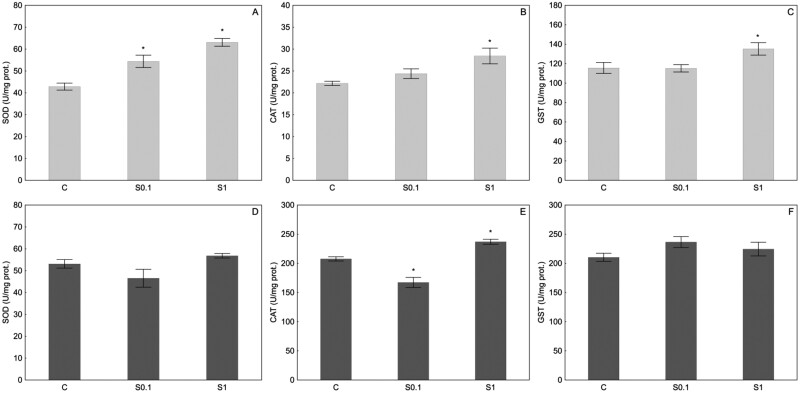
Specific activity (U/mg protein) of superoxide dismutase (SOD), catalase (CAT), and glutathione *S*-transferase (GST) in the head (A, B, and C, respectively) and the abdomen (D, E, and F, respectively) of honey bees whose diet was supplemented with 0.1 mM (S0.1) and 1 mM (S1) spermidine for 17 days. Results are presented as mean ± SEM and analyzed with ANOVA followed by post-hoc Dunnet test. The asterisk (*) on top of the bars denotes significant differences (*P* ≤ 0.05) compared to control (C).

In the present study, the activity of 3 antioxidant enzymes CAT, SOD, and GST was measured in the head and abdomen of honey bee workers, supplemented with 0.1 mM (S0.1) and 1 mM (S1) spermidine for 17 days, and control (C) fed with 50% sucrose. In general, the higher concentration of spermidine, 1 mM, had a stronger effect on the activity of SOD, CAT, and GST, with the effect being more pronounced in the head than in the abdomen of honey bees (Fig. 1).

Spermidine supplementation significantly affected activities of SOD, CAT, and GST in the head of honey bees (*F*_(2,23)_ = 21.26, *P* < 0.001; *F*_(2,23)_ = 6.011, *P* = 0.008; *F*_(2,23)_ = 4.629, *P* = 0.020, respectively). In the head of honey bees supplemented with a higher spermidine concentration (S1 group), the activities of all analyzed enzymes were significantly higher compared to the control (C) (Fig. 1A–C), while in the group supplemented with a lower spermidine concentration (S0.1 group), only the activity of SOD was significantly higher (Fig 1B).

Spermidine supplementation significantly affected activity of CAT and SOD (*F*_(2,24)_ = 34.77, *P* < 0.001 and *F*_(2,24)_ = 3.739, *P* = 0.039, respectively), but not GST (*F*_(2,24)_ = 1.885, *P* = 0.174) in the abdomen of honey bees. In the abdomen, CAT activity was significantly lower in the abdomen of bees fed with a lower spermidine concentration (S0.1 group) and significantly higher in the group fed with a higher spermidine concentration (S1 group) (Fig. 1E). However, no significant change was observed in SOD and GST enzyme activity compared to the control (Fig. 1D, F).

### Relative Expression of Antioxidant Genes

The results of the relative gene expression of the antioxidant defense system enzymes in the head and abdomen are shown in Table 1. More differences were observed in the abdomen than in the head, especially in bees supplemented with lower spermidine concentration (S0.1 group): an upregulation of the genes *Cnc*, *Cat*, and *Sod2*, and a downregulation of *GstS1*. A higher spermidine concentration (S1 group) also led to upregulation of the *Cnc* and *Sod2* genes in the abdomen. In the head, only *Cnc* was upregulated in the S0.1 group, while *Gstmic1* was downregulated. Downregulation of *GstD1* was observed in the S1 group.

**Table 1.** Relative gene expression of Cap’n’collar (*Cnc*), catalase (*Cat*), superoxide dismutases (*Sod1* and *Sod2*), glutathione *S*-transferases (*GstD1*, *GstS1*, *Gstmic1*), prophenoloxidase (*Ppo*), and phenoloxidase-activating factor 2 (*Ppaf2*) in head and abdomen of honey bees supplemented with 0.1 (S0.1) and 1 mM spermidine (S1) for 17 days in comparison with control, fed only with sucrose

		S0.1		S1	
	Gene	Relative expression	SE	Relative expression	SE
**Head**	***Cnc***	**1.4↑**	1.2–2.0	1.4	1.0–1.8
	***Cat***	0.9	0.6–1.0	1.0	0.8–1.2
	***Sod1***	1.2	1.0–1.4	1.0	0.8–1.2
	***Sod2***	1.0	0.9–1.1	0.8	0.7–1.0
	***GstD1***	1.0	0.8–1.1	**0.8↓**	0.7–0.9
	***GstS1***	0.9	0.8–1.0	0.9	0.8–1.1
	***Gstmic1***	**0.8↓**	0.8–0.9	0.9	0.8–1.1
**Abdomen**	***Cnc***	**3.0↑**	2.3–4.0	**2.0↑**	1.5–2.5
	***Cat***	**1.8↑**	1.5–2.5	1.5	1.1–2.0
	***Sod1***	1.0	0.9–1.0	1.1	1.0–1.2
	***Sod2***	**1.7↑**	1.5–1.8	**1.3↑**	1.2–1.5
	***GstD1***	0.9	0.8–1.1	0.9	0.6–1.0
	***GstS1***	**0.7↓**	0.6–0.8	0.9	0.7–1.1
	***Gstmic1***	0.9	0.6–1.2	0.8	0.7–1.0
	***Ppo***	**5.4↑**	3.1–13.4	0.5	0.2–1.1
	***Ppaf2***	**2.9↑**	2.1–s4.0	1.4	1.0–1.9

The range for the standard error (SE) for 68% CI (confidence interval) is presented. The arrow indicates statistically significant gene upregulation (↑) or downregulation (↓) (*P* ≤ 0.05) compared to the control group.

### Phenoloxidase Activity and Gene Expression of *Ppo* and *Ppaf2*

**Fig. 2.**
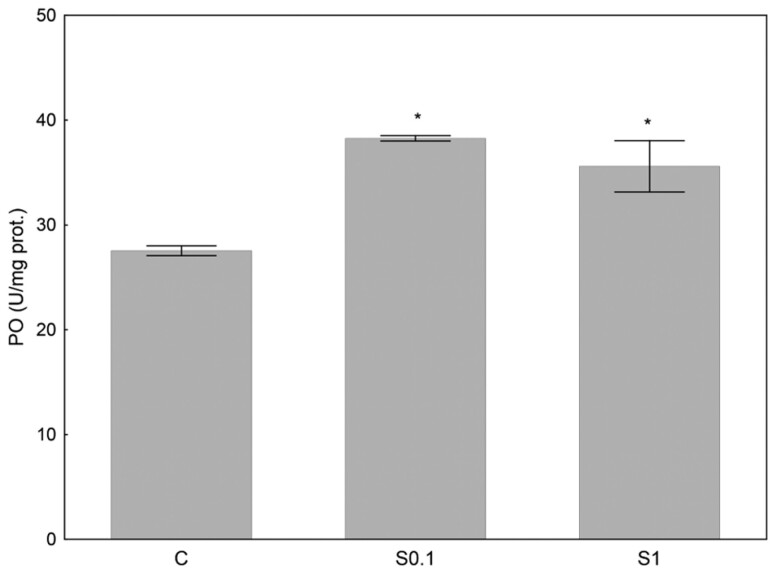
Specific activity of phenoloxidase (PO) (U/mg protein) in the abdomen of honey bees whose diet was supplemented with 0.1 mM (S0.1) and 1 mM (S1) spermidine for 17 days. Results are presented as mean ± SEM and analyzed with ANOVA followed by post-hoc Dunnet test. The asterisk (*) on top of the bars denotes significant differences (*P* ≤ 0.05) compared to control (C).

Phenoloxidase activity was measured in the abdomen of the bees. The activity of this enzyme was significantly higher in both supplemented groups compared to the control (*F*_(2,24)_ = 14.81, *P* < 0.0001) (Fig. 2).

The results of the relative gene expression of the immunity genes *Ppo* and *Ppaf2* in the abdomen are shown in Table 1. Spermidine supplementation increased the expression of the immunity genes *Ppo* and *Ppaf2* in the abdomen of honey bees fed with lower spermidine concentration (S0.1 group).

## Discussion

Bees are important pollinators, but they are currently at risk from various stressors that can cause oxidative stress, which affects their health and lifespan. Dietary supplements that increase antioxidant capacity could help. In a previous study, the use of spermidine supplements at a dosage of 0.1 and 1 mM was found to improve the survival and life expectancy of honey bees (Đorđievski et al. 2023). The same study showed that 17 days of spermidine supplementation reduced oxidative stress and increased antioxidant capacity. Based on this, our study investigated the effects of 17 days of supplementation on specific components of the antioxidative and detoxification system in bees.

The results showed that after supplementing the diet with 0.1 mM spermidine, the increase in the expression of *Cnc*, *Cat*, and *Sod2* genes in the abdomen and *Cnc* in the head suggests a potential role of spermidine in strengthening the antioxidant defense, while the increase in the expression of *Cnc* and *Sod2* genes in the abdomen when supplementing the diet with a higher concentration, 1 mM spermidine, suggests a dose-dependent effect of spermidine on antioxidant pathways. On the other hand, the reduced expression of *Gst* isoforms, particularly *GstS1* in the abdomen and *Gstmic1* in the head when supplemented with a lower concentration, as well as the reduced expression of *GstD1* when supplemented with a higher concentration of spermidine, may indicate a change in detoxification mechanisms, likely due to the induction of autophagy, which may help to remove damaged components that could otherwise generate ROS, potentially reducing the need for detoxification enzymes such as GSTs (Eisenberg et al. 2009). The unchanged expression of the other analyzed genes in the abdomen/head suggests that spermidine cannot directly affect these genes in the mentioned concentrations and tissues. The different gene expression patterns observed in abdominal and head tissues in response to spermidine supplementation suggest tissue-specific and concentration-dependent effects on the expression of important antioxidant and detoxification genes in honey bees. The data of the study by Đorđievski et al. (2023) indicate that almost all of the analyzed key enzymes of polyamine metabolism in the experimental groups supplemented with spermidine showed significantly higher expression levels in the abdomen, but not in the head, supporting this assumption.

When we look at the results of enzyme activity, the increased activity of total SOD in the head (for 0.1 mM spermidine), CAT, total SOD, and total GST in the head, and CAT in the abdomen (for 1 mM spermidine), suggests a potential role of spermidine in enhancing antioxidative capacity. In general, the higher concentration of spermidine, 1 mM, had a greater effect on the measured activity of the antioxidative enzymes. The antioxidative effect of spermidine has been confirmed in previous reports (Eisenberg et al. 2009, Guo et al. 2011, Minois et al. 2012, Xu et al. 2020), but it also could have the pro-oxidative effect as the result of its catabolism, which involves oxidases that produce ROS in the form of H_2_O_2_ (Stewart et al. 2018). Thus, the higher CAT activity in bees supplemented with 1 mM spermidine could be the result of more intense H_2_O_2_ metabolism in both the head and abdomen. In addition, a recent study by Kumar et al. (2022) showed for the first time that the administration of spermidine into the cell interacts with free iron and oxygen to generate the O_2_^−^ radical and increase the Fe^3+^/Fe^2+^ ratio. The higher SOD activity in the head of honey bees supplemented with spermidine could therefore be a prevention against oxidative stress caused by O_2_^−^ that consequently lead to higher production of H_2_O_2_ and stimulation of CAT activity in the head of honey bees. The reduced CAT activity in the abdomen with the addition of 0.1 mM spermidine may appear contradictory to the increased expression of the *Cat* gene. This inconsistency can be attributed to post-transcriptional or post-translational regulatory mechanisms influencing the translation or stability of the CAT enzyme. Furthermore, the GST activity in the head of the bees from the S1 group is also higher compared to the control, which indicates a possible detoxification of polyamine metabolites or because of its role in membrane protection through degradation of lipid hydroperoxides (Agostinelli et al. 1996, Ceci et al. 2017). The unchanged activity of total GST in the abdomen and head (for 0.1 mM spermidine) as well as in the abdomen (for 1 mM spermidine) is consistent with the lack of significant change in the expression of individual isoforms of these genes. Differences in enzyme activity in the head and abdomen could indicate differences in polyamine metabolism in the tissues and organs of the honeybee. The data about the influence of spermidine supplementation in insects are scarce but indicate that activity of SOD and CAT should be measured in separated body parts or isolated organs (Mysarla et al. 2016, Yerra et al. 2016), which is additionally supported by the results presented in this study.

Furthermore, the upregulation of immunity genes *Ppo* and *Ppaf2* (for 0.1 mM spermidine) and increased PO activity (for 0.1 and 1 mM spermidine) in the abdomen indicate that stimulation of immunity by spermidine is regulated at both the transcriptional and post-transcriptional level. At the transcriptional level, the increase in the expression of immune-related genes suggests that spermidine may act on transcription factors or signaling pathways that enhance the transcription of these genes. The lack of upregulation of genes with higher concentrations of spermidine could be due to the fact that they were already upregulated earlier, as gene expression was measured after 17 days of supplementation. The observed increase in phenoloxidase activity, even at different spermidine concentrations, suggests that post-transcriptional mechanisms are also involved, such as increasing translation efficiency and enzyme activation. The ability of spermidine to boost immunity is also observed in other species (Metur and Klionsky 2020, Carriche et al. 2021, Gonzales et al. 2021, Ni and Liu 2021). To our knowledge, this is the first report on the effects of spermidine on non-mammalian immunity. Further research should focus on the study of spermidine supplementation in infected bees to confirm the immunostimulatory effect and to clarify the mechanisms behind this effect.

The observed changes provide a valuable contribution to understanding the molecular mechanisms by which spermidine may exert its beneficial effects on the bee’s health and lifespan. The results are consistent with previous findings on spermidine’s role in extending lifespan and reducing oxidative damage. Integrating enzyme activity data with gene expression results reveals that some changes in enzyme activity are consistent with gene expression, but a direct pattern is not always followed so there are inconsistencies that indicate the involvement of additional regulatory mechanisms. These results further emphasize the importance of considering concentration-dependent effects and tissue-specific responses in understanding the effects of spermidine on bee physiology. Further investigations should consider specific post-transcriptional and post-translational modifications, downstream effects of enzyme activity on the specific signaling pathways, and interactions mediated by spermidine. These results support the idea of the use of spermidine supplementation to promote bee health and resilience to environmental stressors, emphasizing that the dose must be carefully chosen to achieve a balance between the pro- and antioxidant effects of spermidine.

## Supplementary Material

ieae098_suppl_Supplementary_Material

## Data Availability

The data that support the findings of this study are available from the corresponding author upon reasonable request.
