# Supplementary material for: Spermidine supplementation influence on protective enzymes of Apis mellifera (Hymenoptera: Apidae)
Source: J Insect Sci. 2024 Oct 9;24(5):3. doi: 10.1093/jisesa/ieae098 (PMC11462450; doi:10.1093/jisesa/ieae098)
Supplement: ieae098_suppl_Supplementary_Material [file ieae098_suppl_supplementary_material.docx]

**Table S1.** **PCR primer sequences and efficiencies**

| **Amplification target** | | **Sequence** | **Efficiency** | **Reference** |
| --- | --- | --- | --- | --- |
| β-actin (reference gene) | *ActB.F*  *ActB.R* | ATGCCAACACTGTCCTTTCTGG  GACCCACCAATCCATACGGA | 105% | Antúnez et al. (2009) |
| Ribosomal protein 49  (reference gene) | *Rp49.F*  *Rp49.R* | CGTCATATGTTGCCAACTGGT  TTGAGCACGTTCAACAATGG | 88% | Lourenço et al. (2008) |
| Cap'n'collar | *Cnc.F*  *Cnc.R* | GTGGTACAACCTCTTCGGCA  TCCAGTTTCCATCCACTCGC | 92% | NCBI PrimerBlast* |
| Catalase | *Cat.F*  *Cat.R* | GGCGGCTGAATTAAGTGCTA  TTGCGTTGTGTTGGAGTCAT | 96% | Collins et al. (2004) |
| Superoxide dismutase 1 | *Sod1.F*  *Sod1.R* | AGCAGATGCAAGTGGTGTTG  GAGCACCAGCATTTCCTGTAG | 97% | Collins et al. (2004) |
| Superoxide dismutase 2 | *Sod2.F*  *Sod2.R* | GTCGCCAAAGGTGATGTCAATAC  CGTCTGGTTTACCGCCATTTG | 105% | Li et al. (2014) |
| Glutathione S-transferase,  Delta class | *GstD1.F*  *GstD1.R* | GGCTGCCGCTCTCGACATCG  TGCCGTATTGGTCCGCCAAGT | 90% | Morimoto et al. (2011) |
| Glutathione S-transferase,  Sigma class | *GstS1.F*  *GstS1.R* | TGGTGGTGCTCTTTCTTGGGCT  TGTGGACGCTTATCAAGCCAGCT | 99% | Morimoto et al. (2011) |
| Glutathione S-transferase, microsomal | *Gstmic1.F*  *Gstmic1.R* | AATTGCAATGGTTCCATTAACTGCA  ATGTGCTCTGCGTACACGTT | 103% | Morimoto et al. (2011) |
| Prophenoloxidase | *ProPo.F*  *ProPo.R* | TCGGCGATGATACGGAATCG  GTTGACGACGTCCAAGTTGC | 95% | NCBI PrimerBlast* |
| Phenoloxidase-activating factor 2 | *Ppaf2.F*  *Ppaf2.R* | TGGAGAATGGGACACGCAAA  GTTGTAAAGAGTGCCGCCAG | 109% | NCBI PrimerBlast* |

*Primers designed by our group using the NCBI PrimerBlast (Ye et al. 2012)

**References**

Antúnez K, Martín‐Hernández R, Prieto L, et al. 2009. Immune suppression in the honey bee (*Apis mellifera*) following infection by *Nosema ceranae* (Microsporidia). Environ. Microbiol. 11(9): 2284–2290. https://doi.org/ 10.1111/j.1462-2920.2009.01953.x.

Collins AM, Williams V, Evans JD. 2004. Sperm storage and antioxidative enzyme expression in the honey bee, *Apis mellifera*. Insect Mol Biol. 13(2): 141–146. https://doi.org/10.1111/j.0962-1075.2004.00469.x.

Li C, Xu B, Wang Y, et al. 2014. Protein content in larval diet affects adult longevity and antioxidant gene expression in honey bee workers. Entomol. Exp. Appl. 151(1): 19-26. https://doi.org/10.1111/eea.12167.

Lourenço AP, Mackert A, dos Santos Cristino A, et al. 2008. Validation of reference genes for gene expression studies in the honey bee, *Apis mellifera*, by quantitative real-time RT-PCR. Apidologie. 39(3): 372-385. https://doi.org/10.1051/apido:2008015.

Morimoto T, Kojima Y, Toki T, et al. 2011. The habitat disruption induces immune‐suppression and oxidative stress in honey bees. Ecol. Evol. 1(2): 201-217. https://doi.org/10.1002/ece3.21.

Ye J, Coulouris G, Zaretskaya I, et al. 2012. Primer-BLAST: a tool to design target-specific primers for polymerase chain reaction. BMC Bioinformatics. 13: 1-11.
